# Acute renal failure in severe pancreatitis: A population-based study

**DOI:** 10.3109/03009734.2010.547636

**Published:** 2011-04-12

**Authors:** Hung-Yuan Lin, Jiun-I Lai, Yi-Chun Lai, Po-Chou Lin, Gau-Jun Tang, Shih-Chieh Chang

**Affiliations:** ^1^National Yang-Ming University School of Medicine, Taiwan, Republic of China; ^2^Department of Medicine, National Yang-Ming University Hospital, Taiwan, Republic of China; ^3^Shih-Hsin University, Department of Information Management, Taiwan, Republic of China

**Keywords:** Acute renal failure, intensive care, severe acute pancreatitis

## Abstract

**Introduction:**

Acute pancreatitis (AP) is a common illness with varied mortality and morbidity. Patients with AP complicated with acute renal failure (ARF) have higher mortality than patients with AP alone. Although ARF has been proposed as a leading mortality cause for AP patients admitted to the ICU, few studies have directly analyzed the relationship between AP and ARF.

**Methods:**

We performed a retrospective study using the population-based database from the Taiwan National Health Insurance Research Database (NHIRD). In the period from 1 January 2005 to 31 December 2005, every patient with AP admitted to the ICU was included and assessed for the presence of ARF and mortality risk.

**Results:**

In year 2005, there were a total of 221,101 admissions to the ICU. There were 1,734 patients with AP, of which 261 (15.05%) patients also had a diagnosis of ARF. Compared to sepsis and other critical illness, patients with AP had a higher risk of having a diagnosis of ARF, and patients with both diagnoses had a higher mortality rate in the same ICU hospitalization.

**Conclusion:**

AP is associated with a higher risk of ARF, and, when both conditions exist, a higher risk of mortality is present.

## Introduction

Acute pancreatitis (AP) is a relatively common medical illness with a wide range of morbidity and mortality, with an estimated incidence of 35–80 cases per 100,000 each year (1). Severe acute pancreatitis (SAP), as proposed by Bradley, known as the Atlanta classification, is established by either an APACHE II score >8, Ranson score >3 (2), the presence of more than one organ failure, or local complications (3). SAP has been associated with an increased mortality, estimated from 7% to 47% (4,5). Acute renal failure (ARF) in the setting of AP has been shown to have a 10-fold increase in mortality (74.7% versus 7%) in a study of 563 patients (6). A similar result was reported in another study, showing 71.2% mortality versus 6.8% in SAP patients with or without ARF (7). In agreement with the above studies, a recent cross-sectional study reported a 5-fold increase (66.6% with ARF versus 14.5% without ARF) in mortality in SAP patients, providing an updated assessment of the clinical standpoint (8). To our knowledge, few studies directly pin-pointed the relationship between SAP and ARF in ICU patients (8), although both conditions are common etiologies in the ICU and pose diagnostic and therapeutic challenges.

In Taiwan, the National Health Insurance (NHI) program was started in 1995 and covers nearly all inhabitants. Because all claims data of in-patients are available to researchers in electronic form, it is possible to conduct a large-series retrospective study to investigate the prevalence of AP and ARF and related mortality data in ICU patients in Taiwan (9). In light of the above background, we conducted a study using the population-based database of the NHI program in Taiwan, aiming to: 1)assess the prevalence and characteristics of AP patients admitted to the ICU, and 2)identify the prevalence of ARF coexisting with AP, and determine if a relationship exists that can serve as future treatment guide. We then further analyze the statistical significance of the data distribution and identify risk factors leading to increased mortality. This population-based study method is a novel approach in assessment of patients with pancreatitis admitted to ICU, and we anticipate the results to provide clinical significance in treatment planning for intensivists and internists.

## Methods

### Database

Pooled data obtained from the Taiwan National Health Insurance Research Database (NHIRD) in the period from 1 January 2005 to 31 December 2005 were used for analysis in this study. The NHIRD is a nation-wide database including all in-patient medical benefit claims for the Taiwanese population, with an inclusion rate of over 96% of the population. The database includes registries of contracted medical facilities and board-certified physicians, monthly records of in-patient claims summaries, and other in-patient hospitalization details. Each individual operation's procedure codes and diagnosis codes are included with compliance to classification using the International Classification of Disease, 9th revision, Clinical Modification (ICD-9-CM).

This study was approved by the ethics committee of the authors' institution on the basis that no disclosure of any patient's privacy or individual data could be made public due to the encoding nature of the database.

### Study sample

**Figure 1.**
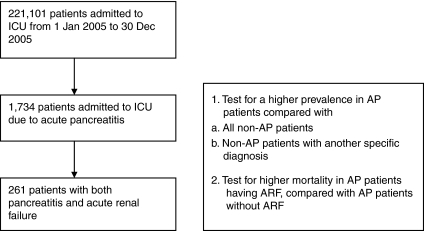
Study design.

We included every ICU hospitalization episode from 1 January 2005 to 31 December 2005. The database included a total of 221,101 patients. We then selected patients with a diagnosis ICD-9 code compatible with acute pancreatitis (ICD-9 code 577.0) and acute renal failure (584.x; x = 0–9). Age, gender, ICD-9 classification codes, and mortality data were recorded (Figure 1).

Each admission episode was recorded for the main diagnosis ICD code using the International Classification of Disease, 9th revision, Clinical Modification (ICD-9-CM). The nature of the claims database was that laboratory information was not included, therefore only the admission date, discharge date, patient profile, and ICD-9 diagnosis codes were available; there was no way of knowing if the coding was correct for each patient. However, according to the National Health Insurance policy, a protocol existed of peer review from another independent physician for the claims. If the diagnosis or claim was found at fault, the claim would be revoked; therefore, an internal validation system exists for the accuracy of each claim reviewed. We rely on this internal control to assume accuracy of our database and the study results.

### Statistical analysis

In this study, MySQL 4.1 (1995–2008 MySQL AB, 2008–2009 Sun Microsystems, Inc.), was used as database software for data linkage and processing. Descriptive data were presented, including number of patients and percentages. Multivariate logistic regression was used to assess hazard ratios and risk (SPSS software, version 14.0, SPSS Corp., Chicago, IL, USA). Results are displayed as coefficients, odds ratios, and 95% confidence intervals. A *P* value ≤ 0.05 (two-tailed) was considered as statistically significant.

## Results

### Statistics of ICU admissions in year 2005

During the period of 1 January 2005 to 31 December 2005, there were a total of 221,101 valid ICU admissions. The gender ratio was male/female: 60.5%/39.5%. The age distribution is summarized in Table I.

**Table I.** Age distribution of ICU admissions in 2005.

Age	Number	Percentage (%)
18 and under	16,571	7.5
19–28	8,685	3.9
29–38	11,831	5.3
39–48	16,599	7.5
49–58	26,140	11.8
59–68	29,984	13.6
69–78	48,533	22.0
Above 78	62,758	28.4
Total	221,101	100

During the period of 1 January 2005 to 31 December 2005, there were a total of 1,734 ICU admissions which included the diagnosis of acute pancreatitis (ICD-9 code:577.0). The gender ratio was male/female: 66.9%/33.1%. The age distribution and mortality rate for each age-group are summarized in Table II.

**Table II.** Age distribution of ICU admissions of acute pancreatitis in 2005.

Age	Number	Prevalence (%)	Mortality rate (%)
18 and under	26	1.5	3.8
19–28	43	2.5	2.3
29–38	179	10.3	7.3
39–48	347	20.0	10.4
49–58	263	15.2	6.1
59–68	211	12.2	8.1
69–78	302	17.4	9.9
Above 78	363	20.9	18.5

### Acute pancreatitis is associated with an increased incidence of acute renal failure

We included every ICU admission to assess patients with diagnosis of acute renal failure (defined as cases with ICD-9 coding of 584.x; x = 0–9), diagnosis of acute pancreatitis (ICD-9 code:577.0), and patients with both diagnoses. Our results showed 261 patients having both diagnosis of AP and ARF (male/female ratio: 74.33%/35.67%). We then compared the probability of ARF occurring in patients with and without AP. Using logistic regression, the incidence of ARF coexisting with AP was 15.05% (261/1734), with an odds ratio of 4.82 when compared to non-AP patients (coefficient value 1.582; odds ratio 4.862; *P* < 0.01).

To avoid confounding factors and to further validate this finding, we separately tested patients with AP against non-AP patients admitted due to another diagnosis for the incidence of ARF. We selected the five leading diagnoses for ICU admission: ischemic heart disease (ICD-9 code: 410–414), lung-related disorders (including respiratory failure) (ICD-9 code: 518), pneumonia (ICD-9 code: 486), cerebrovascular disease (including hemorrhage and infarction of cerebral arteries) (ICD-9 code: 431–434),and sepsis (ICD-9 code: 038). We included non-AP patients who had either one of the above diagnoses, and classified them into five groups according to each diagnosis. We then tested the AP group against each group separately. Using multivariate logistic regression, the AP group showed a significant increase in risk of having a diagnosis of ARF when compared against the other groups (Table III).

**Table III.** Risk of acute renal failure in non-pancreatitis patients with separate diagnoses compared to pancreatitis patients.

	Disease group	Coefficient	Odds ratio	Percentage with acute renal failure	*P* value
1	Ischemic heart disease	−2.213	0.109 (0.089–0.134)	2.5%	<0.05
2	Lung-related disorders (including respiratory failure)	−0.979	0.376 (0.308–0.457)	8.3%	<0.05
3	Pneumonia	−1.081	0.339 (0.278–0.414)	7.3%	<0.05
4	Cerebrovascular disease (including hemorrhage and infarction of cerebral arteries)	−2.493	0.083 (0.066–0.103)	1.9%	<0.05
5	Sepsis	−0.432	0.649 (0.534–0.789)	13.2%	<0.05

Our results show that patients with AP had a higher risk of having a diagnosis of ARF in the same ICU hospitalization, when compared with either all non-AP patients or non-AP patients with an individual diagnosis.

### The presence of acute renal failure is associated with increased mortality in ICU patients admitted due to acute pancreatitis

We assessed whether AP coexisting with ARF was associated with increased mortality. Patients with both diagnosis of AP and ARF had a mortality rate of 23.76% in the same hospitalization when compared to patients with AP alone without ARF, who had a mortality rate of 8.08% (odds ratio 3.752 (2.640–5.331); *P* < 0.05) (Table IV). Mortality was defined as death from any cause in the same course of ICU hospitalization.

**Table IV.** Mortality risk in pancreatitis patients with and without acute renal failure.

	Number	Mortality number	Mortality rate (%)
Patients with pancreatitis and acute renal failure	261	62	23.8
Patients with pancreatitis without acute renal failure	1,473	119	8.1

## Discussion

Severe acute pancreatitis (SAP) in the intensive care unit poses a therapeutic challenge with significant mortality (10). The pathophysiology began as severe systemic inflammatory response in the early stages and necrosis of the pancreas later on (10). It is in the systemic inflammatory process that hypovolemia ensues, either from wide-spread vasodilation or fluid sequestration, with tissue hypoperfusion and ultimately causing organ damage and failure (11). Several studies have described organ failure accompanying pancreatitis, with renal failure always one of the culprit organs (5,10,11). Acute renal failure has been shown to increase mortality in SAP patients (4,5,10), and the progression of acute renal failure should alert the physician of progressive disease.

In our study, a total of 1,734 patients were admitted to the ICU due to acute pancreatitis (0.78%). The prevalentage-groups were age 39–48 (20.01%) and age over 78 (20.93%),which correlated with the risk factors such as increased alcohol consumption in the mid-40s age-group (12). The prevalent age-groups (age 39–48 and age >70) also presented with the highest mortality (10.4% and 18.5%, respectively). The prevalence of acute renal failure coexisting with acute pancreatitis was 15.05%, significantly higher than non-pancreatitis patients. In non-pancreatitis patients, patients with sepsis had the highest incidence of acute renal failure (13.2%). Compared to pneumonia, cerebrovascular disease, myocardial infarction, and respiratory failure, sepsis involves more wide-spread inflammation and organ damage and probably has a more similar pathophysiology with pancreatitis patients with coexisting acute renal failure.

Commonly used prognostic scoring systems for pancreatitis include the Ranson Criteria, APACHE II score, and the Atlanta classification which is usually used for standardizing clinical trials using the above classifications (13). In the Ranson criteria an increase in blood urea nitrogen was indexed as a scoring point, while in the APACHE II system serum creatinine was included as one factor (14). Our study validates this concept that acute renal failure is associated with extremely high mortality in SAP patients, and also shows that severe acute pancreatitis itself is associated with higher incidence of acute renal failure. The exact mechanism for pancreatitis-related renal failure is not yet well settled, but studies have shown that systemic inflammation, cytokine production, free radicals, and other factors influencing microcirculation play a role (15).

In a recent study (8), the investigators assessed multivariate risk factors that would predict outcome. Age, history of kidney disease, and abdominal compartment syndrome were found to be independent prognostic factors (8). The study, using a different approach from that of ours, concluded an increased mortality and duration of ICU stay. The results corroborate our study with its lack of information about patient outcome being there with deprived of definitive predictive power. Combination of that study and ours would provide strong evidence of increased mortality effect of ARF on SAP patients.

Our study provides a novel approach to investigate ICU patients by using a large-scale population-based study. Because of the near universal inclusion of the Taiwanese population in the National Health Insurance, the NHIRD reflects a ‘real world’ database representing statistical significance and near minimal sampling error or selection bias. Our weakness lies in misclassification errors, which is under control owing to the peer review validation of all claims as previously mentioned. Another limitation is that the NHIRD database only includes claims data and relevant treatment data, while laboratory data are not included. This poses a significant limitation to our study that the exact APACHE II score or other scoring system of each patient could not be obtained. We could only rely on the assumption that for a patient to be admitted to the ICU, an APACHE II score of more than 8 should be common; indeed, according to our peer validation, the claim of a patient admitted to the ICU with an APACHE II score less than 8 would very likely be revoked. Therefore, by definition of the Atlanta criteria, patients admitted to the ICU with the diagnosis of acute pancreatitis should classify as severe acute pancreatitis (SAP). Also, the nature of the database precluded the assumption of a causal relationship. However, this study still brings important messages with strong statistical significance owing to its large number of cases and its near total sampling with little room for selection and sampling bias. We can judiciously conclude that acute pancreatitis patients admitted to the ICU (very possibly mostly SAP patients) have a higher risk of having an acute renal failure, and having both diagnoses may lead to higher mortality.

In conclusion, our study of 1,734 patients with acute pancreatitis admitted to the ICU revealed that acute renal failure was more frequently encountered in this critical illness associated with higher mortality. Our results imply that deterioration of renal function in SAP patients should be closely monitored and the possibility of renal damage should be prevented. Our study provides a novel approach to studying ICU patients using a population-based database. Although large case numbers and real-world descriptions are the strengths of our study, further controlled studies with complete clinical characteristics are needed for demonstration of a definite causal relationship.
